# A Novel Spider Toxin Inhibits Fast Inactivation of the Na_v_1.9 Channel by Binding to Domain III and Domain IV Voltage Sensors

**DOI:** 10.3389/fphar.2021.778534

**Published:** 2021-12-06

**Authors:** Shuijiao Peng, Minzhi Chen, Zhen Xiao, Xin Xiao, Sen Luo, Songping Liang, Xi Zhou, Zhonghua Liu

**Affiliations:** The National and Local Joint Engineering Laboratory of Animal Peptide Drug Development, College of Life Sciences, Hunan Normal University, Changsha, China

**Keywords:** Na_v_1.9, fast inactivation, domain III voltage-sensor, spider peptide toxin, neurotoxin receptor site

## Abstract

Venomous animals have evolved to produce peptide toxins that modulate the activity of voltage-gated sodium (Na_v_) channels. These specific modulators are powerful probes for investigating the structural and functional features of Na_v_ channels. Here, we report the isolation and characterization of δ-theraphotoxin-Gr4b (Gr4b), a novel peptide toxin from the venom of the spider *Grammostola rosea.* Gr4b contains 37-amino acid residues with six cysteines forming three disulfide bonds*.* Patch-clamp analysis confirmed that Gr4b markedly slows the fast inactivation of Na_v_1.9 and inhibits the currents of Na_v_1.4 and Na_v_1.7, but does not affect Na_v_1.8. It was also found that Gr4b significantly shifts the steady-state activation and inactivation curves of Na_v_1.9 to the depolarization direction and increases the window current, which is consistent with the change in the ramp current. Furthermore, analysis of Na_v_1.9/Na_v_1.8 chimeric channels revealed that Gr4b preferentially binds to the voltage-sensor of domain III (DIII VSD) and has additional interactions with the DIV VSD. The site-directed mutagenesis analysis indicated that N1139 and L1143 in DIII S3-S4 linker participate in toxin binding. In sum, this study reports a novel spider peptide toxin that may slow the fast inactivation of Na_v_1.9 by binding to the new neurotoxin receptor site-DIII VSD. Taken together, these findings provide insight into the functional role of the Na_v_ channel DIII VSD in fast inactivation and activation.

## Introduction

Voltage-gated sodium (Na_v_) channels are important transmembrane proteins that play a vital role in the generation and propagation of action potentials in excitable cells, such as central and peripheral neurons, cardiac and skeletal muscle myocytes, and neuroendocrine cells (Goldin 2001; Catterall 2012; Mantegazza and Catterall 2012). Nine Na_v_ channels (denoted Na_v_1.1–Na_v_1.9) have been identified in human (Yu and Catterall 2003). The subtypes can be divided into two categories according to their sensitivity to TTX: TTX-sensitive (Na_v_1.1–1.4, Na_v_1.6, and Na_v_1.7) or TTX-resistant (Na_v_1.5, Na_v_1.8, and Na_v_1.9). Notably, these subtypes have different tissue-specific localization and functions. The Na_v_1.1–Na_v_1.3 subtypes are expressed primarily in the central nervous system (CNS); the Na_v_1.6 subtypes are expressed in the central and peripheral nervo us system; the Na_v_1.7–Na_v_1.9 subtypes are mainly expressed in the peripheral nervous system (PNS); Na_v_1.4 is present in skeletal muscle; and Na_v_1.5 is mainly expressed in cardiac muscle (Dib-Hajj et al., 1998; Goldin 2001; Renganathan et al., 2002; Fukuoka et al., 2008; Catterall 2012; Bennett et al., 2019). Structurally, Na_v_ channel consist of an approximately 260 kDa pore-forming α-subunit and one or more associated β-subunits of 30–40 kDa (Catterall 2012; Bennett et al., 2019). The α-subunit has four homologous domains (I–IV). Each domain consists of six transmembrane segments (S1–S6) that form a voltage-sensing domain (VSD) containing S1–S4 and a central pore-forming domain (PD) containing S5, two P-loop, and S6 (Catterall 2000). The 4–8 positively charged arginine or lysine residues at every third position in S4 act as gating charges, which are required for voltage-dependent activation (Numa and Noda 1986; Catterall et al., 2017). The gating charges move outward upon membrane depolarization and initiate the voltage-dependent activation and inactivation of Na_v_ channels (Jiang et al., 2020). These characteristics endow the various conformation transformations of Na_v_ channel via an electromechanical coupling mechanism to open and close the pore. The three major states are defined as resting, activation, and inactivation.

Inactivation, which is an intrinsic property of Na_v_ channels, is a complex process that includes two distinct modes: fast and slow. Fast inactivation involves an inactivation particle in the cytoplasmic linker between DIII and DIV binding to the intracellular side of the pore (Vassilev et al., 1988; Catterall 2012). In contrast, slow inactivation is when the pore domain undergoes conformational rearrangements during prolonged depolarization (Silva and Goldstein 2013). Na_v_ channels undergo fast inactivation on a millisecond timescale to interrupt Na^+^ conductance, which was first described by Hodgkin and Huxley in 1952 (Hodgkin and Huxley 1952). The intracellular loop between DIII and DIV forms the fast inactivation gate in which the three hydrophobic amino acids, namely Ile, Phe, and Met (IFM motif), are the key sequence (West et al., 1992; Catterall 2012). The cryo-EM structure of eukaryotic Na_v_ channels shows a potential allosteric blocking mechanism for fast inactivation (Yan et al., 2017). The IFM motif plugs into the compact hydrophobic pocket formed by the S4–S5 linker of DIII and DIV and the intracellular ends of S5 and S6 of DIV (McPhee et al., 1994, 1995, 1998; Kellenberger et al., 1996; Smith and Goldin 1997; Jiang et al., 2020). Although the mechanism of the development of fast inactivation is unclear, we believe that the fast inactivation allosteric process requires voltage-sensing and electromechanical coupling, which may involve a contribution from one or more VSD to cause the conformational changes. Fluorescent labeling studies have shown that the VSDs in DI-DIII of the Na_v_1.4 channel are activated by depolarization faster than in DIV, which is consistent with the time course of activation and fast inactivation (Cha et al., 1999; Chanda and Bezanilla 2002; Kubota et al., 2017). This suggests that the activation gate opening of the Na_v_ channel is in contact with the outward movement of voltage sensors in DI-DIII, whereas fast inactivation is initiated by subsequent movement of the voltage sensor in DIV. Moreover, the known α-scorpion toxins, which inhibit the outward movement of DIV VSD to prevent DIV activation, slow the fast inactivation of Na_v_ channels (Campos et al., 2008; Clairfeuille et al., 2019). These findings demonstrate that DIV initiates fast inactivation of Na_v_ channels. However, whether other domain VSDs participate in the development of fast inactivation remains unknown, although evidence to date suggests that is not the case.

Venomous animals (spiders, scorpions, cone snails, etc.) have evolved the ability to produce peptide toxins with high affinity to target Na_v_ channels for the capture of prey or enhanced defenses against predators (Stevens et al., 2011). These peptide toxins (also known as neuropeptide toxins) are useful pharmacological tools for exploring the physiological roles of Na_v_ channels and a potentially rich source for drug discovery. The interactions between these toxins and Na_v_ channels can occur in two different ways: by occluding pores (pore blockers) or altering gating kinetics (gating modifier toxins). At least three distinct binding sites of neuropeptide toxins have been identified (Stevens et al., 2011). Peptide toxins binding to site 1 use the first mechanism, e.g., some μ-conotoxins from cone snails are site 1 pore blockers. Site 1 is mainly localized in the extracellular loops between S5 and S6 of DI–DIV (Chau et al., 2011). Site 3 toxins, like α-toxins (from scorpion, spider and sea anemone), slow fast inactivation and bind to the S3–S4 extracellular loop in domain IV (Thomsen and Catterall 1989; Catterall et al., 2007). Site 4 peptide toxins (β-toxins) regulate activation kinetics by binding to the extracellular loop connecting the S3-S4 segments in DII (Catterall et al., 2007; Xiao et al., 2008; Song et al., 2011; Zhang et al., 2020). It is known that the central pore of Na_v_ channel-mediated ion flow and the DII VSD is associated with channel activation and the DIV VSD is responsible for fast inactivation of channels. Thus, these peptide toxins can serve as pharmacological tools to provide insight into the structural and functional features of Na_v_ channels.

In this study, we identified and characterized the spider peptide toxin Gr4b from the venom of the spider *Grammostola rosea*, which is a gating modifier and significantly inhibits fast inactivation of the Na_v_1.9 channel. Like the previously described Na_v_1.9 peptide toxin HpTx1 (Zhou et al., 2020), Gr4b also inhibits the currents of Na_v_1.4 and Na_v_1.7 but does not affect Na_v_1.8. Interestingly, Gr4b displays a novel effect on Na_v_1.9, which occurs mostly through binding to the DIII S3-S4 linker to slow fast inactivation. This is distinct from HpTx1 which only binds to the DIV S3-S4 linker. Thus, the results of our study provide direct evidence for the role of DIII VSD in Na_v_ channel fast inactivation and provide a new tool to probe the structural and functional features of Na_v_ channels.

## Materials and Methods

### Venom Collection and Toxin Purification

As described in previous studies, the venom of the *Grammostola rosea* spider was collected by electrical stimulation. The collected venom was lyophilized and stored at −80°C. Subsequently, the venom was dissolved in 0.1% trifluoroacetic acid (TFA) in double-distilled water to a final concentration of 10 mg/ml immediately before being subjected to reversed-phase high-performance liquid chromatography (RP-HPLC) purification. First, reverse-phase HPLC purification was performed using a water HPLC system (Waters Alliance, 2695 HPLC system) with an Ultimate^®^ XB-C18 column (10 × 250 mm, 5 μm, Welch Materials Inc., Shanghai, China) with a flow rate of 3 ml/min and a gradient of 10–55% A for more than 45 min (solvent A: 0.1% trifluoroacetic acid in acetonitrile, solvent B: 0.1% trifluoroacetic acid in water). The absorbance was measured at 215 nm. The fractions were collected, lyophilized, and then stored at –20°C until the next subdivision. Next, the target fraction containing Gr4b was subjected to a second round of RP-HPLC (Waters Alliance, 2695 HPLC system) using an analytic XB-C18 column, (300 Å, 4.6 mm × 250 mm, Welch Materials Inc., Shanghai, China) with a linear increasing acetonitrile gradient (acetonitrile at an increasing rate of 0.5% per minute and a flow rate of 1 ml/min) to obtain the purified Gr4b. The molecular weight of the peptide was confirmed by matrix assisted laser desorption/ionization-time of-flight mass-spectrometry (MALDI-TOF-TOF MS) spectrometry (AB SCIEX TOF/TOFTM 5800 system, Applied Biosystems, United States). The N-terminal amino acid sequence of the peptide was determined by automated Edman degradation in a PPSQ-53A protein sequencer (Shimadzu Corporation, Kyoto, Japan).

### Plasmid Constructs and Mutagenesis

Rat Na_v_1.4, human Na_v_1.7 and rat Na_v_1.8 cDNA clones were kindly gift from Dr. Theodore Cummins (Stark Neurosciences Research Institute, Indiana University School of Medicine, Indianapolis, IN, United States) and were subcloned into the pCMV or pCDNA3.1-blank vectors. Human Na_v_1.9 was subcloned into the pEGFP-N1 vector. The C-terminal of hNa_v_1.9 was linked to GFP to construct a fusion protein channel (hNa_v_1.9-eGFP), which was as described in our previous studies (Zhou et al., 2017). Mutations were made using the site-directed mutation method or recombination-based cloning using GenBuilder™ Cloning Kit (GenScript, United States). Primers presented in Supplementary Tables 1–4. All mutations were verified by DNA sequencing.

### Cell Culture and Transfection

ND7/23 and HEK293T cells were maintained at 37°C in a humidified 5% CO_2_ incubator in Dulbecco’s Modified Eagle’s Medium (DMEM) supplemented with 10% fetal bovine serum, 100 μg/ml streptomycin, 100 U/ml penicillin, and 2 mM L-glutamine. The cells were trypsinized, diluted with 1 ml of culture medium, and seeded at a 1:5 ratio in 35 mm Petri dishes for culture. When grown to 80–90% confluence, the ND7/23 cells were transfected with hNa_v_1.9-GFP or hNa_v_1.9-GFP mutants using the X-tremeGENE HP DNA Transfection Reagent (Roche, Basel, Switzerland) according to the manufacturer’s instructions. ND7/23 cells were used for hNa_v_1.9-GFP chimeric channel expression and the conditions were as previously described (Zhou et al., 2017), the beta subunits (β1 and β3) are endogenously expressed in the cell lines used to study Na_v_1.9 (Rogers et al., 2016). Transfections for the other plasmids were performed using Lipofectamine 2000 (Invitrogen, Carlsbad, CA, United States) following the manufacturer’s instructions. Na_v_ channel plasmid (4 µg) plus 0.5 µg pEGFP-N1 (except for hNa_v_1.9) plasmid were co-transfected into HEK293T or ND7/23 (rNa_v_1.8 and hNa_v_1.9 only) cells. The cells were seeded onto several 3.5 cm dishes at a 1:10 ratio at 4–6 h after transfection. Cells with green fluorescent protein (GFP) were selected for whole-cell patch-clamp analysis at 24–36 h post-transfection.

### Electrophysiology Recordings

Whole-cell current recordings were performed using an EPC-10 USB patch-clamp amplifier operated by Patch Master software (HEKA Elektronik, Lambrecht, Germany). The recording pipettes were fabricated from borosilicate glass capillaries using a two-step vertical microelectrode PC-10 puller (Narishige Group, Tokyo, Japan), and the pipette resistance was controlled to be 2.0–3.0 MΩ. Voltage-clamp recordings were acquired with Patch Master software 2 × 73 (HEKA Elektronik) 4 min after establishing whole-cell configuration, and the currents elicited were sampled at 20 kHz and filtered at 5 kHz. After breaking in, the serial resistance was controlled below 5 MΩ, the voltage error was minimized by using 80% serial resistance compensation, and the compensation speed value was 10 µs. For recording Na_v_ channel currents, the external solution contained (mM): 150 NaCl, 2 KCl, 1.5 CaCl2, 1 MgCl2 and 10 HEPES (pH 7.4, adjusted with NaOH); the pipette solution contained (in mM): 35 NaCl, 105 CsF, 10 EGTA, 10 HEPES (pH 7.4, adjusted with CsOH). The osmotic pressure of the intracellular fluids and extracellular fluids is adjusted to 300–320 mOsm with sucrose. Before use, Gr4b was dissolved in ddH_2_O to make a 250 μM stock solution at −20°C. TTX was dissolved in DMSO to make a 1 mM stock solution. TTX was added to bath solution to a final concentration of 1 µM when used to inhibit TTX-sensitive (TTX-S) Na_v_ channels. Unless otherwise indicated, all chemicals were products of Sigma-Aldrich (St. Louis, MO, United States). For electrophysiology experiments, the stock solution of Gr4b was diluted with fresh bath solution to a concentration of tenfold of the interested concentration, 30 µl of the concentrated peptide was diluted into the recording chamber (containing 270 µl bath solution) far from the recording pipet (the recording cell), and was mixed by repeatedly pipetting to achieve the specified final concentration.

### Data Analysis

Data were analyzed using the PatchMaster v2x73 (HEKA Elektronik, Lambrecht, Germany), Igor Pro 6 (Wave Metrics, Lake Oswego, OR, United States), Office Excel 2010 (Microsoft Corporation, WA, United States), and GraphPad Prism 7 (GraphPad Software Inc., CA, United States). All data points are shown as mean ± standard error of the mean (SEM), and n was presented as the number of separate experimental cells. The Boltzmann function was used to fit steady-state activation and deactivation curves. Concentration-response curves were fitted using the Hill equation. One-way ANOVA was used to assess the difference between multiple groups. Significant levels were set at *p* < 0.05.

## Results

### Toxin Purification and Identification

**FIGURE 1:**
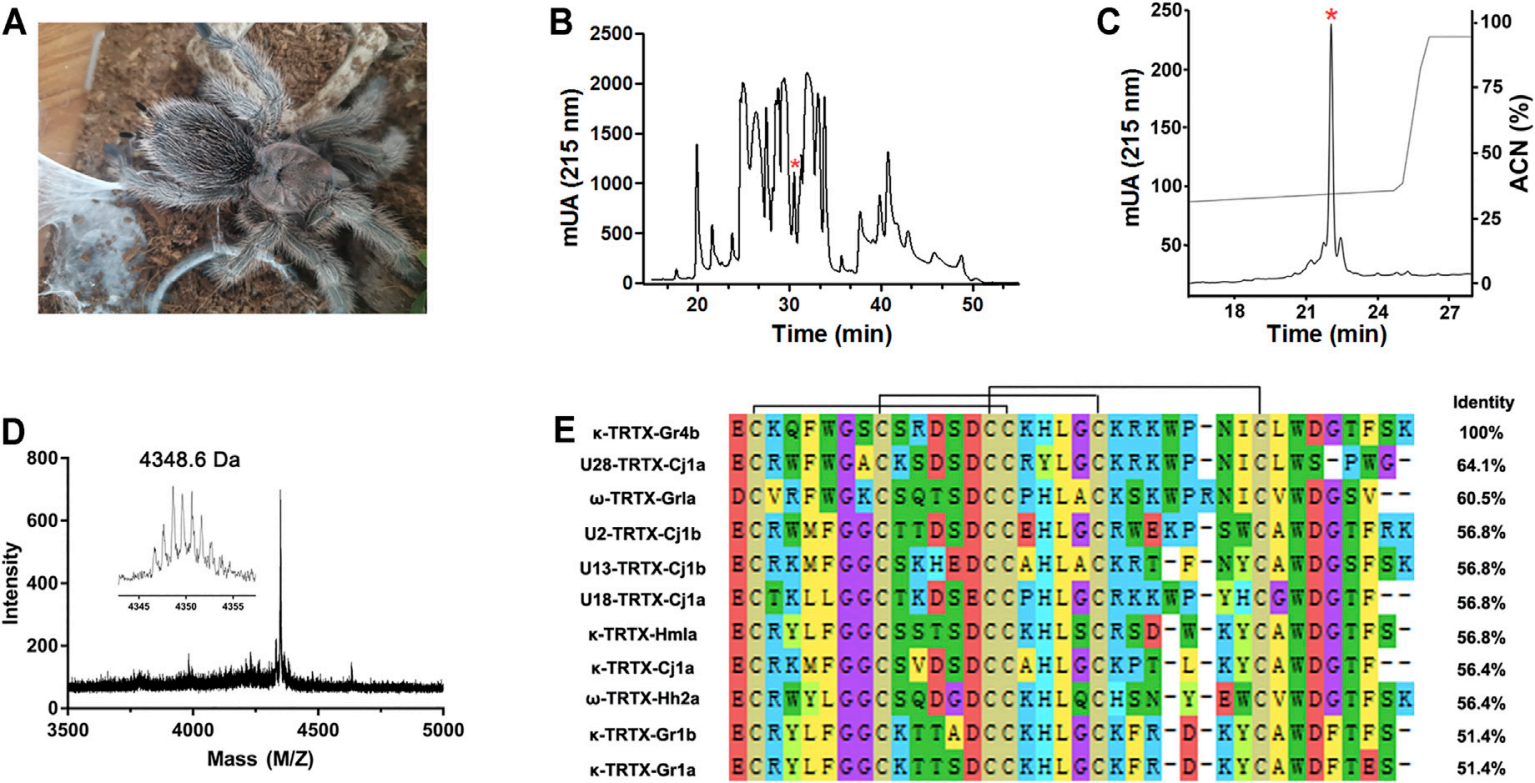
Purification and identification of Gr4b. **(A)** The spider G. *Grammostola rosea.* **(B)** RP-HPLC profile of crude venom from spider *Grammostola rosea*. The asterisk indicated the peak containing Gr4b. **(C)** Gr4b was purified to homogeneity by analytical RP-HPLC. **(D)** MALDI-TOF MS spectrum showing a monoisotopic M+H^+^ of 4348.6 Da. **(E)** Sequence alignment of Gr4b with similar toxins in NaSpTx family2; black lines show the disulfide linkage.

The venom of the spider *Grammostola rosea* contains several classes of peptide toxins that target ion channels (Figure 1A). For example, GpTx1 and PaurTx3 are potent inhibitors of the Na_v_1.7 channel (Murray et al., 2015; Chen et al., 2020), HaTx1 and VsTx1 significantly inhibit the currents of the K_v_2.1 channel (Chen et al., 2012; Bemporad et al., 2006), and GsMTx2/4 blocks mechanosensitive ion channels (Oswald et al., 2002). Due to poor heterologous expression in mammal cells, pharmacological studies of the Na_v_1.9 channel have lagged. Previously, we succeeded in achieving functional expression of the Na_v_1.9 channel in heterologous cells (Zhou et al., 2017). Using this system, we first identified a spider peptide toxin that activates the Na_v_1.9 channel and produces pain in mice (Zhou et al., 2020). In order to identify more specific and novel peptide toxins for Na_v_1.9, we used patch-clamp recording to screen animal peptide toxins for the ability to affect the Na_v_1.9 channel. Crude venom was fractionated by RP-HPLC, as shown in Figure 1B. By screening the panel, venom fractions with significant Na_v_1.9 regulation activity were identified; a fraction potently inhibited the fast inactivation of Na_v_1.9 (Figure 1B). This fraction was further purified by RP-HPLC, and approximately 8 μg of purified peptide toxin was obtained from 1 mg crude spider venom (Figure 1C). The purity of this peak was confirmed by MALDI–TOF MS analysis which revealed a peptide toxin with a molecular weight of 4,348.6 Da, which was consistent with the calculated molecular mass (4,348.01 Da). (Figure 1D). N-terminal Edman sequencing and the venom gland transcriptome cDNA data determined a novel 37-residue peptide toxin named Gr4b (rational nomenclature: δ-theraphotoxin Gr4b) (Kimura et al., 2012), as shown in Figure 1E. Sequencing alignment showed that Gr4b shares highly sequence similarity with Family 2 Na_v_-targeting spider toxins (NaSpTx), which comprise 42–44 residues and contained six residues and form a conserved cysteine pattern-inhibitor cystine knot (ICK) motif (Klint et al., 2012) (Figure 1E). NaSpTx Family 2 toxins have various ion channel activities that inhibit K_v_, Ca_v_, and Na_v_ channels (Klint et al., 2012). Interestingly, the members of the NaSpTx Family 2 Toxins JZTX-XI and Df1a display dual modulatory effects on specific Na_v_ channels, simultaneously inhibiting peak current and slowing fast inactivation (Liao et al., 2006; Klint et al., 2012; Tang et al., 2014; Cardoso et al., 2017).

### Effect of Gr4b on Na_v_ Channel Subtypes

**FIGURE 2:**
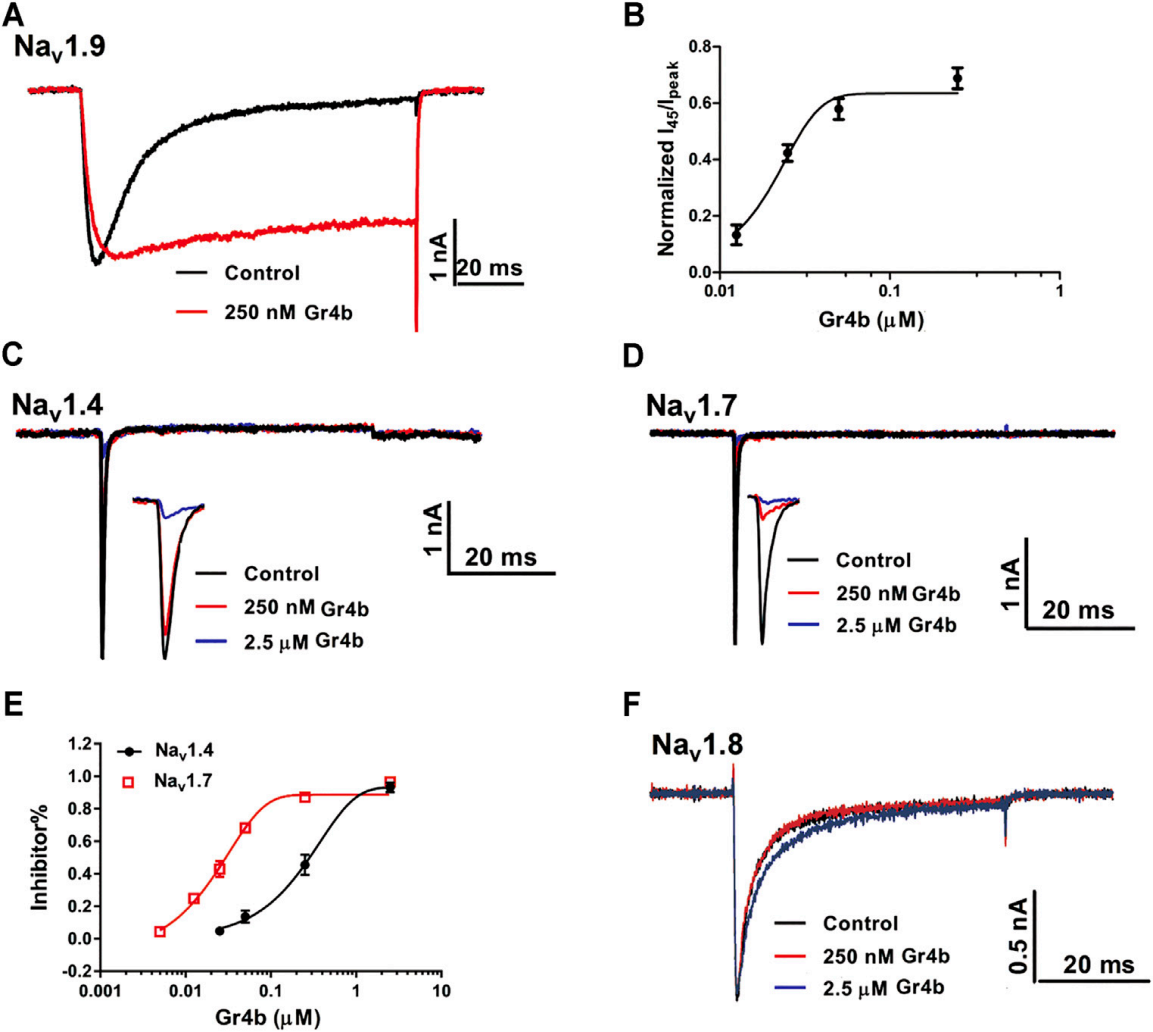
Effect of Gr4b on Na_v_ channel subtypes. **(A)** Representative current traces showing that Gr4b slowed the fast inactivation of Na_v_1.9 expressed in ND7/23 cells. The currents evoked by 100 ms depolarization to −40 mV from holding potentials of −120 mV. **(B)** The dose-response curves for the Gr4b-induced inhibition of the fast inactivation of Na_v_1.9, EC_50_ of 25 ± 1 nM (n = 6). **(C,D)** Representative current traces show that Gr4b blocked the currents of Na_v_1.4 and Na_v_1.7. Currents were elicited by 50 ms depolarizing steps to −10 mV from a holding potential of −90 mV. **(E)** Dose-response curves for Gr4b inhibiting Na_v_1.4 and Na_v_1.7 currents. The IC_50_ values were 411 ± 184 nM and 27 ± 3 nM for hNa_v_1.4 and Na_v_1.7, respectively (n = 4–6). **(F)** Representative traces show that the Na_v_1.8 currents are unaffected by Gr4b (n = 4). Representative current traces from ND7/23 cells expressing Na_v_1.8 in the absence (black) and presence of 250 nM (red) or 2.5 μM (blue) Gr4b. Currents were elicited by 50 ms depolarizing steps to 10 mV from a holding potential of −90 mV.

The Na_v_1.9 current was evoked to −40 mV by a 100-ms depolarization potential from a holding potential of −120 mV in ND7/23 cells. A concentration of 250 nM Gr4b slowed the fast inactivation of Na_v_1.9, leading to a large sustained current (Figure 2A). The half-maximum effective concentration (EC_50_) of Gr4b was determined to be 25 ± 1.0 nM (Figure 2B). Next, we evaluated the effect of Gr4b on a range of Na_v_ channels expressed in HEK293T or ND7/23 cells. Interestingly, Gr4b inhibited Na_v_1.4 and Na_v_1.7 channels in HEK293T cells, with preference for Na_v_1.7 (Figures 2C–E). As shown in Figures 2D,E, 250 nM Gr4b completely inhibited Na_v_1.7 currents with a half-maximum inhibition concentration (IC_50_) value of 27 ± 3.0 nM. It had substantially weaker effects on Na_v_1.4, with an IC_50_ value of 411 ± 184 nM (Figures 2C,E). However, application of up to 2.5 μM Gr4b had no effect on Na_v_1.8 subtype in ND7/23 cells (Figure 2F). Taken together, these results suggest that Gr4b has different actions on different Na_v_ channel subtypes.

### Effect of Gr4b on Activation and Inactivation of Na_v_1.9

**FIGURE 3:**
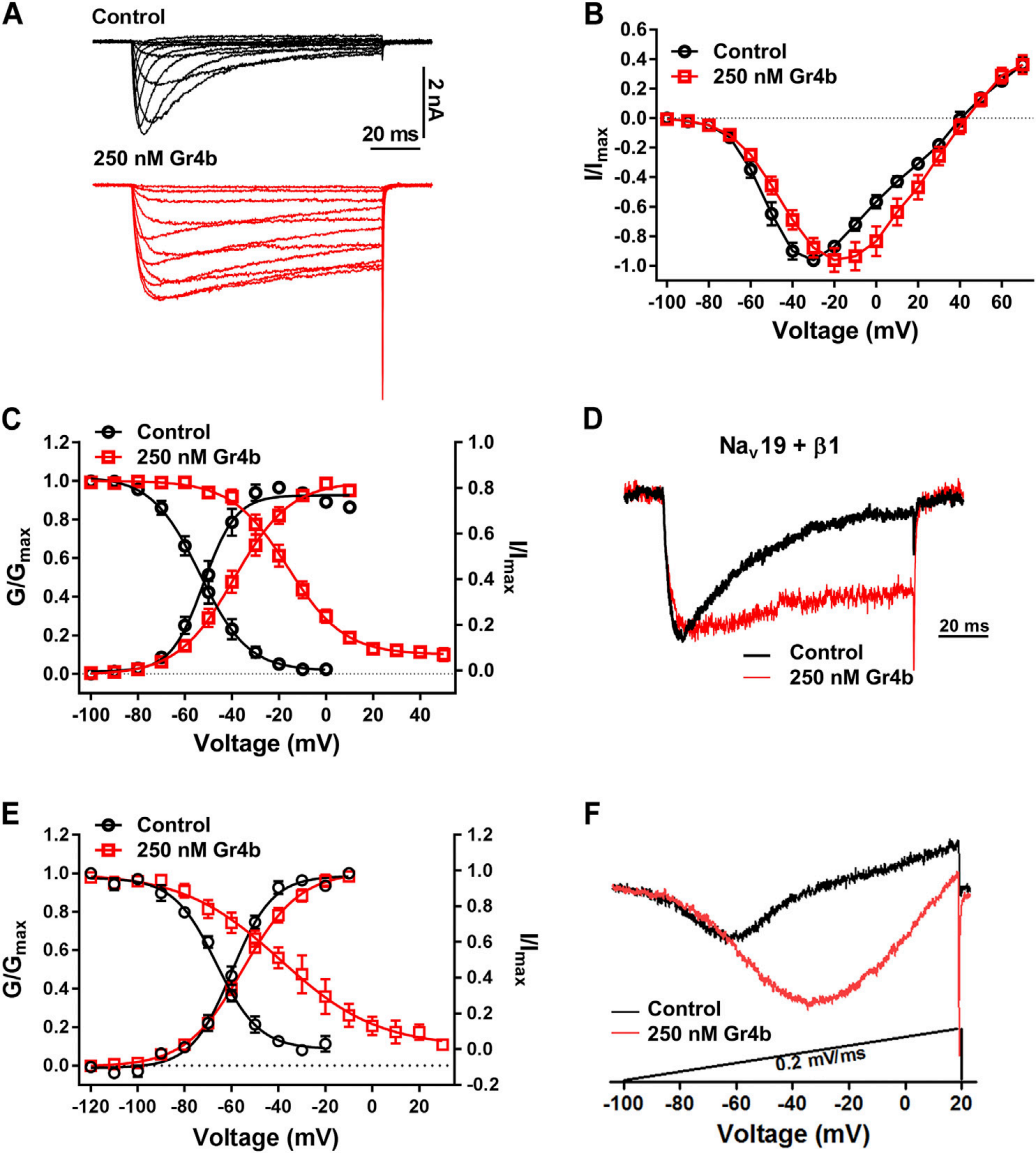
Effect of Gr4b on activation and inactivation of Na_v_1.9. **(A)** Representative current traces of Na_v_1.9 tested by different voltages, in the absence (black) and presence of 250 nM Gr4b (red). **(B)** Current-voltage (I–V) curves for the Na_v_1.9 channel in the absence (black) or presence (red) of 250 nM Gr4b (n = 8). **(C)** Voltage-dependent steady-state activation (G/G_max_, n = 8) and fast inactivation (I/I_max_, n = 6) of Na_v_1.9 in the absence (black) or presence (red) of 250 nM Gr4b. Currents were elicited by a cluster of depolarizations from −100 mV to +70 mV (in 10 mV increments) from the holding potential of −120 mV for 50 ms. For simplicity, only part of the currents were shown. The voltage dependence of steady-state inactivation was estimated by using a standard double-pulse protocol, in which a 50 ms depolarizing test potential to −30 mV followed a 500 ms prepulse (ranged from −120 mV to +50 mV, in 10 mV increment). **(D)** Representative current traces showing that Gr4b slowed the fast inactivation of Na_v_1.9 co-expressed with β1 in ND7/23 cells. The currents evoked by 100 ms depolarization to −40 mV from holding potentials of −120 mV. **(E)** Voltage-dependent steady-state activation (G/G_max_, n = 5) and fast inactivation (I/I_max_, n = 6) of Na_v_1.9 co-expressed with β1 in the absence (black) or presence (red) of 250 nM Gr4b. **(F)** Compared with control treatment, 250 nM Gr4b significantly enhances the ramp currents of Na_v_1.9 channels expressed in ND7/23 cells.

A major effect of gating modifier toxins on Na_v_ channels is modification of the voltage dependence of channel activation and inactivation. Gr4b plays the role of a gating modifier toxin to slow fast inactivation of the Na_v_1.9 channel. To clarify the mode of action of Gr4b, we used the saturation concentration (250 nM) to analyze the effects of Gr4b on the voltage dependence of activation and inactivation properties of the Na_v_1.9 channel. As shown in Figure 3A, Gr4b inhibited the fast inactivation currents at all tested voltages, but did not change the threshold of the initial activation voltage or the reversal potential of the Na_v_1.9 current (Figure 3B). However, the peak of the current was significantly shifted by +10 mV (Figure 3B). In addition, Gr4b shifted the voltage dependence of the activation curve to a more positive potential by approximately 12.5 mV (Control: −51.3 ± 2.7 mV, Gr4b: −38.8 ± 2.9 mV, n = 8, *p* < 0.0001) (Figure 3C and Table 1). Furthermore, a remarkable change was observed in the slopes of the curves from 6.3 ± 0.3 mV in the control to 11.2 ± 0.4 mV in the presence of Gr4b (Figure 3C and Table 1, *p* < 0.0001), indicating that toxin binding might affect the cooperativity of the four voltage sensors of the Na_v_1.9 channel. As shown in Figure 3C and Table 1, the steady-state inactivation curve was significantly shifted to a positive direction by approximately 37.1 mV in the presence of 250 nM Gr4b (Control: −53.7 ± 2.2 mV, Gr4b: −16.6 ± 2.7 mV, n = 6, *p* < 0.0001), whereas the slope of the curve was not changed (Control: 10.0 ± 0.8 mV, Gr4b: 11.3 ± 0.7 mV, n = 6). We also found that Gr4b introduced a non-inactivated component in the steady-state inactivation curve around the test potential. The β1 subunit is known to modulate the kinetics of fast inactivation (Vijayaragavan et al., 2001; Vijayaragavan et al., 2004). Because Gr4b affects inactivation kinetics, we tested whether overexpression of the β1 with Na_v_1.9 altered the effect of the toxin. As shown in Figures 3D,E and Table1, Gr4b significantly inhibited the fast inactivation currents of Na_v_1.9 co-expression with β1 in ND7/23 cells, and shifted the kinetics of fast inactivation and activation to positive potential, similar to that of the effect of toxin on Na_v_1.9 expression in ND7/23 cells. The predicted window currents of the Na_v_1.9 channel were obviously improved in the presence of 250 nM Gr4b (Figure 3C). Indeed, 250 nM Gr4b robustly increased the peak of the ramp current of Na_v_1.9 currents in ND7/23 cells by 42.40% (Control: −404.0 ± 116.3 pA, Gr4b: −701.1 ± 236.6 pA, n = 5, *p* < 0.05) (Figure 3F and Table 2). Consistent with the effect of Gr4b on activation, the peak of the ramp current was observably shifted by 21.9 mV (Control: −47.4 ± 1.5 mV, Gr4b: −25.6 ± 3.4 mV, n = 5, *p* < 0.001), potentially increasing Na^+^ influx (Figure 3F and Table 2). Based on these findings, Gr4b is clearly a gating modifier that alters the voltage dependence of activation and inactivation of the Na_v_1.9 channel.

**TABLE 1:** The effects of Gr4b on activation and inactivation of Na_v_1.9.

	Control						250 nM Gr4b					
	Voltage dependence of Activation (mV)			Voltage dependence of Inactivation (mV)			Voltage dependence of Activation (mV)			Voltage dependence of Inactivation (mV)		
	V_1/2_	k	n	V_1/2_	k	n	V_1/2_	k	n	V_1/2_	k	n
Na_v_1.9	−51.3 ± 2.7	6.3 ± 0.3	8	−53.7 ± 2.2	10.0 ± 0.8	6	−38.8 ± 2.9^****^	11.2 ± 0.4^****^	8	−16.6 ± 2.7^****^	11.3 ± 0.7	6
Na_v_1.9 + *β*1	−60.0 ± 1.3	9.3 ± 1.5	5	−63.2 ± 4.4	10.0 ± 0.9	5	−54.3 ± 0.9^**^	13.4 ± 1.9^***^	5	−38.9 ± 6.6^*^	23.1 ± 4.0^*^	5

Data are presented as the mean ± SEM. ^**^ *p* < 0.01, ^***^ *p* < 0.001, ^****^ *p* < 0.0001. Parametric paired two-tailed *t*-test was used. n is presented as the number of the separate experimental cells.

**TABLE 2:** The effects of Gr4b on ramp current of Na_v_1.9.

	Control				250 nM Gr4b			
	Voltage of the peak current (mV)	n	The peak current of ramp (pA)	n	Voltage of the peak current (mV)	n	The peak current of ramp (pA)	n
Na_v_1.9	−47.4 ± 1.5	5	−404.0 ± 116.3	5	−25.6 ± 3.4^***^	5	−701.1 ± 236.6	5

Data are presented as the mean ± SEM. ^*^ *p* < 0.05, ^***^ *p* < 0.001, when compared with Control. Parametric paired two-tailed *t*-test was used. n is presented as the number of the separate experimental cells.

### Kinetics of Dissociation of Gr4b From Na_v_1.9

**FIGURE 4:**
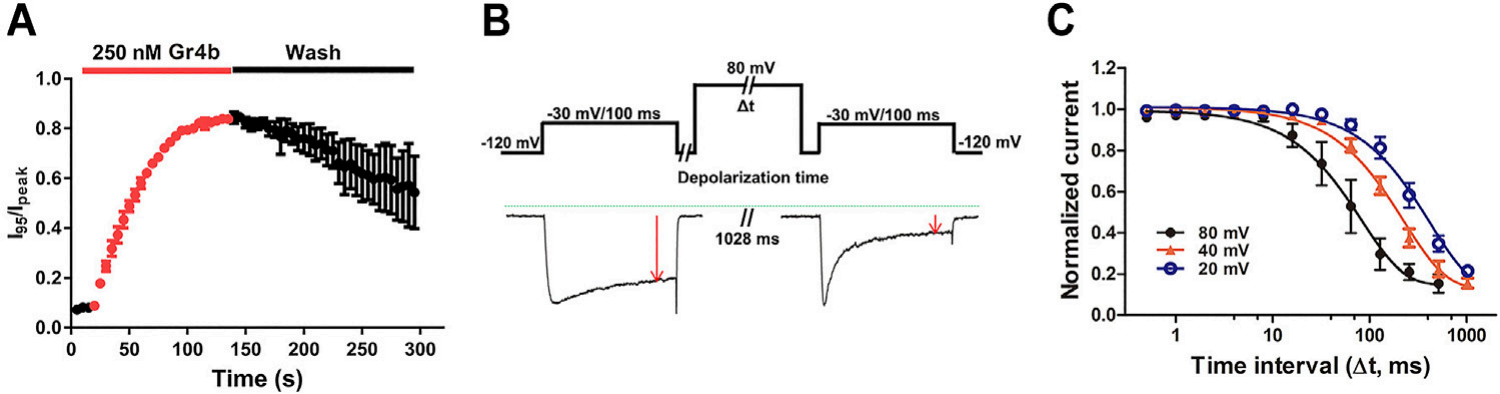
Kinetics of dissociation of Gr4b from Na_v_1.9. **(A)** Time course of the inhibition of fast inactivation by 250 nM Gr4b and the recovery upon washing with bath solution, in which the ratios of I_95_/I_peak_ were plotted as a function of time, τ_on_ = 42.8 ± 3.5 s (n = 3). **(B)** Diagram showing the protocol for dissociation analysis (**upper**). Representative current showing in the presence of 0.25 μM Gr4b, progressively prolonged strong depolarization (to 80 mV) led to a greater degree of Na_v_1.9 current recovery from Gr4b inhibition (**below**). **(C)** Time course of dissociation of 250 nM Gr4b from Na_v_1.9 at 80 mV (τ = 105.6 ± 36.3 ms, n = 4), 40 mV (τ = 240.0 ± 26.9 ms, n = 4), and 20 mV (τ = 473.5 ± 90.8 ms, n = 4). Cells expressing Na_v_1.9 were incubated in Gr4b for 110 s at a holding potential of −120 mV to allow binding. The rate of toxin dissociation was determined with the illustrated pulse paradigm by stepping to a depolarizing pulse of 80, 40, or 20 mV for 0–1,028 ms, returning to −120 mV for 100 ms to allow recovery from fast inactivation, and then assessing the effect of the depolarizing pulse with a 100 ms test pulse to −30 mV. Data are presented as the mean ± SEM.

We further investigated the binding kinetics of toxin on the Na_v_1.9 channel. As shown in Figure 4A, the time course for 250 nM Gr4b inducing the inhibited inactivation currents of Na_v_1.9 was characterized by a slow onset of action, with a τ_on_ value of 42.8 ± 3.5 s (n = 3). Inhibition of fast inactivation by Gr4b slowed the reversible recovery upon perfusion bath solution washing, with a recovery of approximately 54.39% of the control current within 2.5 min (Figure 4A). These results indicate that Gr4b has a strong affinity for the Na_v_1.9 channel.

Gating modifier toxins typically affect channel gating by regulating the voltage-sensor of the channel. In turn, the affinity of the toxins to the channel is also regulated by the stimulus voltage, e.g., the spider toxin ProTx-II inhibits the current of the Na_v_1.7 channel with a significant voltage dependence (Xiao et al., 2010). We previously reported that binding of the gating modifier toxins HNTX-III, HpTx1, and HWTX-IV was reversed by prolonged strong depolarizations that activate the voltage sensor (Xiao et al., 2008; Xiao et al., 2010; Zhou et al., 2020; Liu et al., 2013). Therefore, we examined whether prolonged strong depolarizations could reverse the inhibitory effect of Gr4b on Na_v_1.9 using the protocol described in Figure 4B. As shown in Figure 4B, a progressively longer strong depolarization (up to 80 mV) led to an increase in the fraction of sodium current recovered from inhibition by Gr4b. A depolarization time lasting 1,028 ms resulted in complete recovery of the slowed inactivated current. These data indicate that Gr4b dissociated from Na_v_1.9 in response to prolonged strong depolarizations. Furthermore, as shown in Figure 4C, the degree of dissociation is positively related to the depolarization time and the depolarization voltage, i.e., increased depolarization time and potential were correlated with increased dissociation. From the time course of dissociation of the toxin after strong depolarizations in the presence of 250 nM Gr4b, the dissociation time constant was fitted with a single exponential function and calculated to be 105.6 ± 36.3 ms, 240.0 ± 26.9 ms, and 473.5 ± 90.8 ms for 80 mV, 40 mV, and 20 mV (n = 4 each), respectively. These results suggest that the rate of Gr4b dissociation is voltage-dependent and that stronger depolarization is correlated with a higher rate of dissociation.

### Gr4b Inhibits Fast Inactivation of Na_v_1.9 via the VSD of DIII and DIV

**FIGURE 5:**
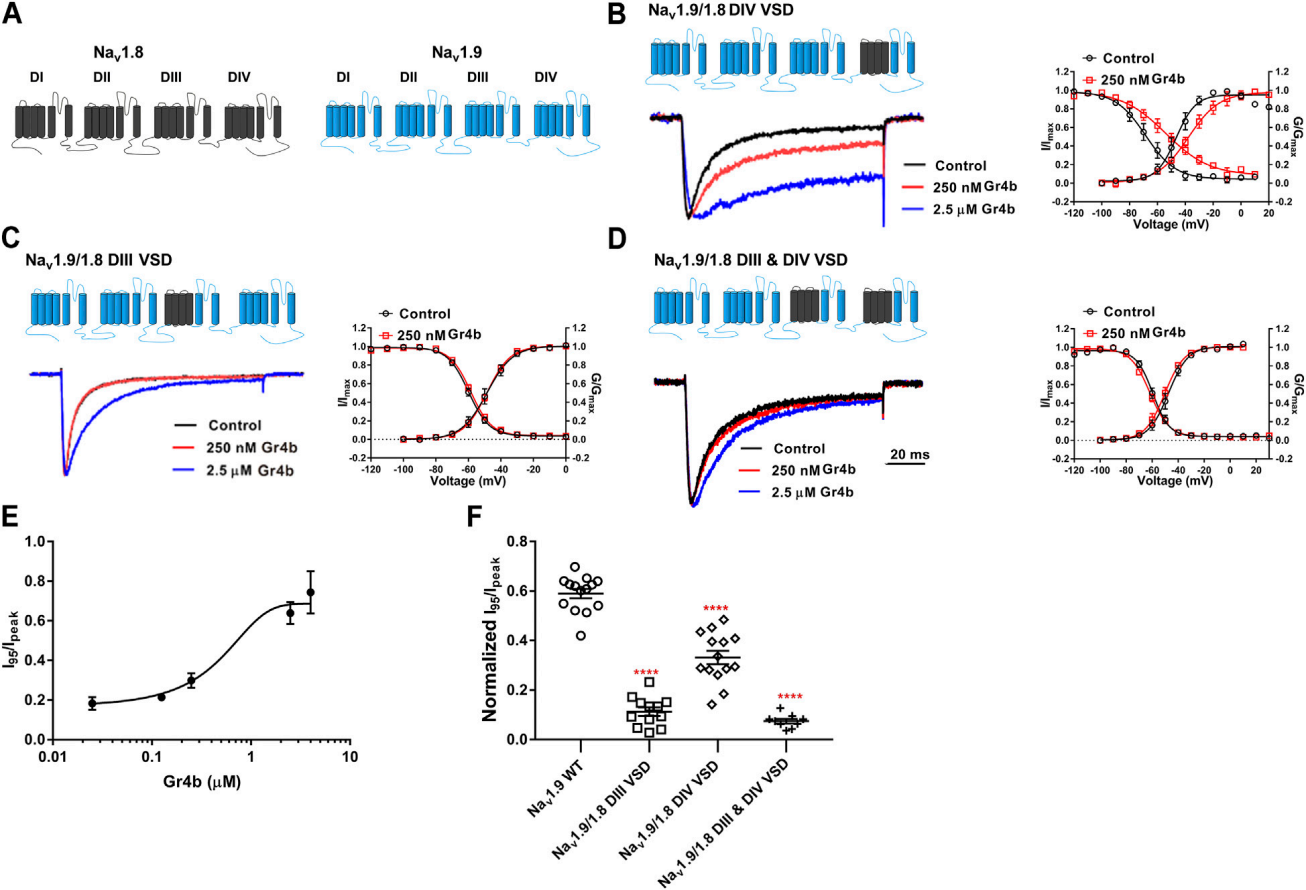
DIII VSD and DIV VSD of Na_v_1.9 is involved in interacting with Gr4b. **(A)**The topological structure of Na_v_1.9 (blue) and rNa_v_1.8 (grey), the numbers indicated the border of the transmembrane domains. **(B–D)** Representative normalized current traces, voltage-dependent steady-state activation and voltage-dependent steady-state inactivation show that the chimera channels (constructed as the cartoon illustrated) Na_v_1.9/1.8 DIV VSD, Na_v_1.9/1.8 DIII VSD, Na_v_1.9/1.8 DIII & DIV VSD, in the absence (black), presence of 250 nM Gr4b (red) or 2.5 μM Gr4b (blue). **(E)** The dose-response curves for the Gr4b-induced inhibition of the fast inactivation of Na_v_1.9/1.8 DIV VSD, EC_50_ of 1.5 ± 0.25 μM (n = 4). **(F)** The effect of Gr4b on the channel of WT and Na_v_1.9/1.8 chimeras. Dot plots display the effect of 250 nM Gr4b on the persistent current (I_95_/I_peak_) (n = 14 for WT; n = 12 for Na_v_1.9/1.8 DIII VSD, n = 14 for Na_v_1.9/1.8 DIV VSD and n = 9 for Na_v_1.9/1.8 DIII & DIV VSD). One-way ANOVA with Dunnett’s Multiple Comparison Test and compared with WT, ^****^ *p* < 0.0001.

Because Gr4b dissociation from Na_v_1.9 is voltage-dependent, we hypothesized that the toxin may be bound to the VSD of the channel. Neurotoxins that act on Na_v_ channels can target six different sites in the channels, with site 3 (DIV VSD) being the hotspot for spider peptide toxins to inhibit fast inactivation (Stevens et al., 2011). One well-characterized example of a Na_v_1.9 modulating peptide is HpTx1 from spider venom, which inhibits fast inactivation of the Na_v_1.9 channel by binding to the DIV S3–S4 linker (Zhou et al., 2020). In the present study, the underlying mechanism of action of Gr4b on Na_v_1.9 channels was similar to that described for HpTx1. To identify the critical region of Na_v_1.9 for toxin-induced inhibition of fast inactivation, several chimeric channels were constructed. Since Na_v_1.8 is resistant to Gr4b, a chimera strategy was used to screen the critical modules (VSD) responsible for the toxin’s ability to reduce fast inactivation of Na_v_1.9. Firstly, we made the chimera Na_v_1.9/1.8 DIV VSD, in which the DIV VSD (DIV S1–S4) of Na_v_1.9 was replaced with the corresponding domain of Na_v_1.8. As shown in Figure 5B, compared with the wildtype (WT) channel, we observed that a Gr4b concentration of 250 nM reduced the efficacy of the Na_v_1.9/1.8 DIV VSD chimeric channel (Figures 5B,F). But the steady-state activation curve was significantly shifted to a positive direction by approximately 8 mV in the presence of 250 nM Gr4b (Control: −45.8 ± 3.4 mV, Gr4b: −37.8 ± 3.5 mV, n = 3, *p* < 0.05) and the slope of the curve was also significantly changed (Control: 6.9 ± 0.6 mV, Gr4b: 10.7 ± 1.4 mV, n = 3, *p* < 0.05) (Table 3), that consistent with the effect of Gr4b on WT channel. Nevertheless, it still produced large inhibition of fast inactivation of the chimera channel at 2.5 μM, similar to that of WT-Na_v_1.9. However, at this concentration, the toxin did not seem to affect Na_v_1.8. Therefore, these results indicate that the channel DIV VSD might be involved in the Gr4b–Na_v_1.9 interaction to inhibit fast inactivation and that additional binding areas may exist.

**TABLE 3:** The effects of 250 nM Gr4b on activation of Na_v_1.9 mutants.

	Control						250 nM Gr4b					
	Voltage dependence of Activation (mV)			Voltage dependence of Inactivation (mV)			Voltage dependence of Activation (mV)			Voltage dependence of Inactivation (mV)		
	V_1/2_	k	n	V_1/2_	k	n	V_1/2_	k	n	V_1/2_	k	n
Na_v_1.9	−51.3 ± 2.7	6.3 ± 0.3	8	−53.7 ± 2.2	10.0 ± 0.8	6	−38.8 ± 2.9****	11.2 ± 0.4****	8	−16.6 ± 2.7****	11.3 ± 0.7	6
Na_v_1.9/1.8 DIII VSD	−48.6 ± 2.5	7.3 ± 0.5	5	−59.8 ± 1.3	6.3 ± 0.4	6	−48.4 ± 1.0	7.0 ± 0.4	5	−58.5 ± 1.0	6.4 ± 0.5	6
Na_v_1.9/1.8 DIV VSD	−45.8 ± 3.4	6.9 ± 0.6	3	−67.9 ± 3.8	10.0 ± 0.4	6	−37.8 ± 3.5	10.7 ± 1.4	3	−54.7 ± 4.1^**^	13.7 ± 0.7^**^	6
Na_v_1.9/1.8 DIII&DIV VSD	−47.7 ± 2.3	7.3 ± 0.4	3	−59.5 ± 1.6	6.0 ± 0.4	5	−50.6 ± 2.1^*^	7.3 ± 0.4	3	−61.7 ± 1.3	7.3 ± 0.6^**^	5
Na_v_1.9/1.8 DIII S3-S4	−55.0 ± 2.4	6.3 ± 0.5	4	−66.0 ± 3.4	7.3 ± 0.5	4	−57.9 ± 2.2	7.4 ± 0.7^*^	4	−65.4 ± 3.9	7.5 ± 0.2	4

Data are presented as the mean ± SEM. ^*^ *p* < 0.05, ^**^ *p* < 0.01, ^***^ *p* < 0.001, ^****^ *p* < 0.0001, when compared with Control. Parametric paired two-tailed *t*-test was used. n is presented as the number of the separate experimental cells.

**FIGURE 6:**
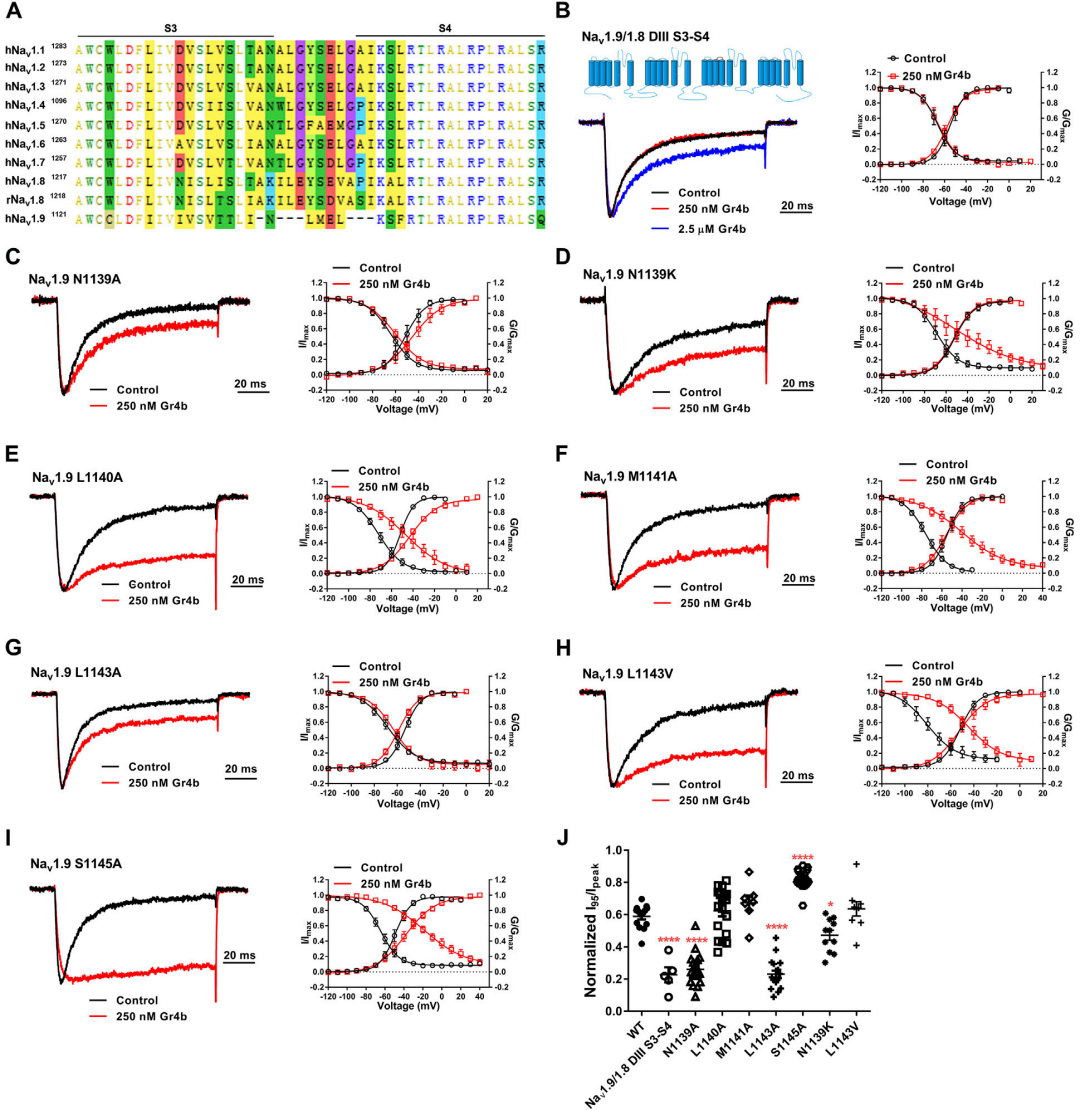
DIII S3-S4 of Na_v_1.9 is involved in interacting with Gr4b. **(A)** Sequence alignments corresponding to Na_v_ subtype domains III (DIII) S3-S4. **(B)** Representative normalized current traces, voltage-dependent steady-state activation and voltage-dependent steady-state inactivation show that the chimera channels (constructed as the cartoon illustrated) Na_v_1.9/1.8 DIII S3-S4, in the absence (black), presence of 250 nM Gr4b (red) or 2.5 μM Gr4b (blue). **(C–I)** Representative traces, voltage-dependent steady-state activation and voltage-dependent steady-state inactivation show that the single point mutations of Na_v_1.9, in the absence (black) and presence of 250 nM Gr4b (red). **(J)** Effects of Gr4b on WT and mutant Na_v_1.9 channels. Dot plots display the effect of 250 nM Gr4b on the persistent current (n = 17 for N1139A, n = 11 for N1139K, n = 18 for L1140A and L1143A, n = 7 for M1141A, n = 9 for L1143V, n = 20 for S1145A and n = 5 for Na_v_1.9/1.8 DIII S3-S4). One-way ANOVA with Dunnett’s Multiple Comparison Test and compared with WT, ^*^ *p* < 0.05, ^****^ *p* < 0.0001.

The DIII VSD of Na_v_ channels have been also shown to modulate channel inactivation (Hsu et al., 2017). Thus, we hypothesized that DIII VSD also is critical for Gr4b inhibition of fast inactivation of Na_v_1.9. Thus we made the two chimeras Na_v_1.9/1.8 DIII VSD and Na_v_1.9/1.8 DIII & DIV VSD. The results showed that replacing these regions of Na_v_1.9 abolished the effects of Gr4b on the channel. Even at high concentrations (2.5 μM), the chimeric channels were almost unaffected by Gr4b (Figures 5C,D,F). However, the EC_50_ of Gr4b was determined to be 1.5 ± 0.25 μM in Na_v_1.9/1.8 DIV VSD channel (Figure 5E). These results suggest that the DIII VSD of the Na_v_1.9 channel plays a key role in Gr4b-mediated inhibition of their fast inactivation currents. Furthermore, we observed that replacing DIII S3-S4 linker region of Na_v_1.9 abolished the effects of Gr4b on the channel also (Figures 6A,B,J). To further elucidate the mechanism underlying Gr4b binding to Na_v_1.9, amino acid residues in the S3–S4 linker of DIII were replaced by the alanine residues (Figure 6A). The results indicated that three component residues (namely N1139, L1143, and S1145) were critical for Gr4b binding to DIII S3-S4. Mutations in N1139 and L1143 significantly reduced sensitivity to Gr4b (Figures 6B–J and Table 4). In addition, a mutation of Na_v_1.9 (S1145A) enhanced sensitivity to Gr4b (Figures 6I,J and Table 4). Taken together, these results suggest that Gr4b preferentially binds to the DIII VSD and has additional interactions with the DIV VSD, while two residues (N1139 and L1143) in the DIII S3-S4 linker may affect the interaction of Na_v_1.9 with Gr4b.

**TABLE 4:** The effects of 250 nM Gr4b on activation of Na_v_1.9 mutants.

	Control						250 nM Gr4b					
	Voltage dependence of Activation (mV)			Voltage dependence of Inactivation (mV)			Voltage dependence of Activation (mV)			Voltage dependence of Inactivation (mV)		
	V_1/2_	k	n	V_1/2_	k	n	V_1/2_	k	n	V_1/2_	k	n
Na_v_1.9 N1139A	−51.5 ± 3.8	7.2 ± 1.0	5	−63.9 ± 3.0	9.5 ± 0.5	6	−43.8 ± 4.5^**^	12.9 ± 2.1^*^	5	−60.6 ± 2.8	11.9 ± 0.6^*^	6
Na_v_1.9 N1139K	−51.1 ± 2.3	8.8 ± 0.9	8	−68.5 ± 3.9	9.6 ± 0.5	4	−52.1 ± 1.8	8.8 ± 0.7	8	−49.7 ± 13.0	19.6 ± 2.7^*^	4
Na_v_1.9 L1140A	−52.1 ± 0.9	6.3 ± 0.2	5	−71.3 ± 2.8	10.5 ± 0.9	7	−42.6 ± 1.6^**^	13.2 ± 1.2^**^	5	−45.0 ± 7.1^**^	15.6 ± 0.8^**^	7
Na_v_1.9M1141A	−54.7 ± 2.7	7.2 ± 0.5	5	−76.3 ± 2.7	9.9 ± 0.2	5	−55.5 ± 2.8	10.3 ± 2.0	5	−37.6 ± 7.1^**^	21.9 ± 1.0^***^	5
Na_v_1.9 L1143A	−53.1 ± 1.8	6.7 ± 0.4	6	−68.3 ± 3.4	10.9 ± 0.3	5	−58.7 ± 1.8^*^	8.1 ± 5.0^**^	6	−63.5 ± 2.2^*^	10.3 ± 0.6	5
Na_v_1.9 L1143V	−51.9 ± 2.2	7.2 ± 0.2	6	−79.9 ± 4.8	11.8 ± 0.4	4	−50.8 ± 3.9	11.4 ± 1.3^*^	6	−44.3 ± 3.8^**^	14.9 ± 1.8	4
Na_v_1.9 S1145A	−49.1 ± 2.6	7.4 ± 0.4	7	−63.9 ± 2.5	8.8 ± 0.4	8	−37.8 ± 4.1^**^	15.0 ± 1.6^**^	7	−17.0 ± 5.0^****^	20.1 ± 2.1^**^	8

Data are presented as the mean ± SEM. ^*^ *p* < 0.05, ^**^ *p* < 0.01, ^***^ *p* < 0.001, ^****^ *p* < 0.0001, when compared with Control. Parametric paired two-tailed *t*-test was used. n is presented as the number of the separate experimental cells.

## Discussion

Spider venoms comprise complex mixtures of various chemical substances, the majority of which are small, disulfide-rich peptides. These bioactive peptides are rich in Na_v_ channel modulators useful for predation and defense. Given their specificity and high affinity for Na_v_ channels, some of these peptides have become useful pharmacological tools for investigating the structure of the Na_v_ channel and studying the activation and inactivation processes that are a fundamental gating characteristic of Na_v_ channels. In this study, we screened the novel spider toxin Gr4b, which is a gating modifier that specifically delays fast inactivation of Na_v_1.9. Gr4b belongs to NaSpTx Family 2 and contains a conserved ICK motif. Analyses to identify the site of action revealed that Gr4b preferentially interacts with the DIII VSD of the Na_v_1.9 channel and, to a relatively lesser extent, with the DIV VSD. These results imply that, similar to the function of DIV VSD, DIII VSD may regulate fast inactivation.

Furthermore, structural and mutational studies revealed that the DIII S4–S5 linker is a docking receptor for the fast inactivation gate IFM. The IFM motif is responsible for fast inactivation via penetration of a compact hydrophobic pocket formed by the S4–S5 linker from DIV, the S4–S5 linker from DIII, and the intracellular ends of S5 and S6 from DIV (Yan et al., 2017; Jiang et al., 2020; Kellenberger et al., 1996; McPhee et al., 1994, 1995; Smith and Goldin 1997). Thus, the deactivation of voltage-gated sodium channels is closely related to DIII and DIV. However, based on the electromechanical coupling mechanism of the sodium channel, the motion of the S4 segment is coupled with the S4–S5 linker to the intracellular activation gate to open the pore. Thus, DIII S4-induced shifts of the DIII S4-S5 linker may be helpful for exposing the docking site of the fast inactivation gate IFM. Furthermore, we found that Gr4b binds to the DIII VSD of Na_v_1.9 and impedes movement in depolarization, resulting in the suppression of fast inactivation. This conclusion is based on the following observations: (1) the effect of Gr4b on the Na_v_1.9 channel is voltage-dependent and the relationships of the current-voltage curve and voltage-dependent steady-state activation curve both shift in the direction of depolarization (Figures 3B,C); and (2) toxin dissociation from the Na_v_1.9 channel was voltage-dependent and time-dependent, and the inhibition of fast inactivation was abolished during long-term depolarization (Figure 4C). Moreover, voltage-clamp fluorescent recordings to observe the Na_v_1.4 VSDs revealed that DIII and DIV VSD immobilization is correlated with the onset of inactivation (Cha et al., 1999); some mutants in DIII VSD were shown to impair fast inactivation and cause channelopathies, e.g., a mutation of R1135H in DIII S4 of Na_v_1.4 significantly enhanced entry into inactivation and prolonged recovery to cause hypokalemia periodic paralysis (Groome et al., 2014). Together, these findings suggest that the DIII VSD of the Na_v_ channel plays a prominent role in regulating inactivation.

The DIII VSD of the Na_v_ channel might be a neurotoxin binding site. To date, at least six different neurotoxin receptor sites have been identified on Na_v_ channels (Klint et al., 2012); however, there are no prior reports of neurotoxins binding to DIII VSD. In the present study, our results indicated that Gr4b preferentially binds to the DIII S3-S4 linker of the Na_v_1.9 channel, and two residues (N1139 and L1143) in the DIII S3-S4 linker of Na_v_1.9 might be involved in the interaction with Gr4b (Figure 6). Previously reported DIV VSD binding toxins, like the α-scorpion toxin LqqIV and spider toxins HpTx1 and Hm1a, have a common feature that shifts the steady-state inactivation curve to more positive potentials and produces a non-inactivated component in the steady-state inactivation curve (Bosmans and Tytgat 2007; Osteen et al., 2017; Zhou et al., 2020). In contrast, DIV VSD binding toxins do not change the steady-state activation curve but enhance the peak current. We found that Gr4b significantly shifts the activation curve to the depolarization direction and weakly suppresses the current of Na_v_1.9 at voltages of −60 to −40 mV (Figures 3B,C). These effects distinguish this toxin from other DIV VSD binding toxins. Notably, these effects were limited to the channels where DIII VSD of Na_v_1.9 exists (Figures 5B–D). The chimeric channels (19/18DIII VSD and 19/18DIII VSD & DIV VSD) abolished the effects of Gr4b, suggesting that the effect of Gr4b on channel activation depends on the toxins binding to DIII VSD. Taken together, our results suggest that the interaction of the toxin with Na_v_1.9 DIII VSD affects fast inactivation of the channel as well as activation.

In summary, our study has revealed a novel spider peptide toxin that specifically interacts with the Na_v_1.9 channel, as well as a novel Na_v_ channel neurotoxin binding to the site DIII VSD. The toxin binding to DIII VSD of Na_v_1.9 affects both activation and fast inactivation, thereby providing pharmacological insight into the role of the DIII VSD in Na_v_ channel activation and fast inactivation.

## Data Availability

The original contributions presented in the study are included in the article/Supplementary Material, further inquiries can be directed to the corresponding authors.
