# Supplementary material for: A Novel Spider Toxin Inhibits Fast Inactivation of the Nav1.9 Channel by Binding to Domain III and Domain IV Voltage Sensors
Source: Front Pharmacol. 2021 Dec 6;12:778534. doi: 10.3389/fphar.2021.778534 (PMC8685421; doi:10.3389/fphar.2021.778534)
Supplement: Supplementary file 1 [file Table1.docx]

**Supplementary Table 1 | Primers used in this study to construct Na_v_1.9/1.8 DIII VSD chimaeras.**

| **Primer name** | **Sequence (5’-3’)** |
| --- | --- |
| rNa_v_1.8 DIII VSD For | AAAACCTGCTACCAAATCGTGGAGCACAGCTGGTTT |
| rNa_v_1.8 DIII VSD Rev | CACCTTCATTCCTTCGAATCGAGACAGAGCCCG |
| hNa_v_1.9 DIII VSD M For | TTCGAAGGAATGAAGGTGGTGGTC |
| hNa_v_1.9 DIII VSD M Rev | CACGATTTGGTAGCAGGTTTTCCG |
