# Supplementary material for: A Novel Spider Toxin Inhibits Fast Inactivation of the Nav1.9 Channel by Binding to Domain III and Domain IV Voltage Sensors
Source: Front Pharmacol. 2021 Dec 6;12:778534. doi: 10.3389/fphar.2021.778534 (PMC8685421; doi:10.3389/fphar.2021.778534)
Supplement: Supplementary file 3 [file Table3.docx]

**Supplementary Table 3 | Primers used in this study to construct Na_v_1.9/1.8 DIII S3-S4 chimaeras.**

| **Primer name** | **Sequence (5’-3’)** |
| --- | --- |
| Na_v_1.9/1.8 S3-S4 8 For | TCTGTGACCACCCTCATTGCGAAGATCCTTGAGTATTCCGACCGGACTCTACGAGCA |
| Na_v_1.9/1.8 S3-S4 8 Rev | AATGAGGGTGGTCACAGAGACAAT |
| Na_v_1.9/1.8 S3-S4 7 For | CTTGAGTATTCCGACGTGGCGTCCATCAAAGCCCTTCGGACTCTACGAGCA |
| Na_v_1.9/1.8 S3-S4 7 Rev | GTCGGAATACTCAAGGAT |
