# Supplementary material for: A Novel Spider Toxin Inhibits Fast Inactivation of the Nav1.9 Channel by Binding to Domain III and Domain IV Voltage Sensors
Source: Front Pharmacol. 2021 Dec 6;12:778534. doi: 10.3389/fphar.2021.778534 (PMC8685421; doi:10.3389/fphar.2021.778534)
Supplement: Supplementary file 4 [file Table4.docx]

**Supplementary Table 4 | Primers used in this study to construct Na_v_1.9 point mutations.**

| **Primer name** | **Sequence (5’-3’)** |
| --- | --- |
| Na_v_1.9 N1139A For | ACCACCCTCATTGCCTTAATGGAA |
| Na_v_1.9 N1139A Rev | AATGAGGGTGGTCACAGAGACAAT |
| Na_v_1.9 N1139K For | ACCACCCTCATTAAATTAATGGAATTGAAG |
| Na_v_1.9 N1139K Rev | AATGAGGGTGGTCACAGAGACAAT |
| Na_v_1.9 L1140A For | CTCATTAACGCAATGGAATTGAAG |
| Na_v_1.9 L1140A Rev | GTTAATGAGGGTGGTCACAGA |
| Na_v_1.9 M1141A For | CTCATTAACTTAGCGGAATTGAAGTCC |
| Na_v_1.9 M1141A Rev | TAAGTTAATGAGGGTGGTCACAGA |
| Na_v_1.9 L1143A For | TTAATGGAAGCGAAGTCCTTCCGGACT |
| Na_v_1.9 L1143A Rev | TTCCATTAAGTTAATGAG |
| Na_v_1.9 L1143V For | AACTTAATGGAAGTGAAGTCCTTCCGGACT |
| Na_v_1.9 L1143V Rev | TTCCATTAAGTTAATGAGGGT |
| Na_v_1.9 S1145A For | GAATTGAAGGCCTTCCGGACTCTA |
| Na_v_1.9 S1145A Rev | CTTCAATTCCATTAAGTT |
